# Anterior Cruciate Ligament Reconstruction: Common Intraoperative Mistakes and Techniques for Error Recovery

**DOI:** 10.1007/s12178-025-09947-w

**Published:** 2025-02-04

**Authors:** Kevin C. Wang, Timothy Keeley, Drew A. Lansdown

**Affiliations:** Department of Orthopaedic Surgery, University of California San Francisco, San Francisco, CA USA

**Keywords:** Anterior cruciate ligament, ACL, ACL reconstruction, Tunnel blowout

## Abstract

**Purpose of Review:**

Anterior cruciate ligament (ACL) reconstruction is a commonly performed procedure among general orthopedists, and is a logged procedure required for graduation from accredited orthopaedic residency programs.

**Recent Findings:**

ACL reconstruction surgery has a number of critical steps, and intraoperative errors can significantly impact the success rate and morbidity of this operation. Technical errors are frequently cited as some of the most common reasons for ACL reconstruction failure. This narrative review provides low-volume surgeons and trainees with an overview of the common errors that can be made during the critical steps of an ACL reconstruction procedure.

**Summary:**

We suggest technical points for avoiding commonly-encountered errors and provide a description of evidence-supported error recovery techniques to address these errors if they occur intraoperatively. These key steps include femoral tunnel creation, tibial tunnel creation, graft harvest and preparation, and graft fixation within the tunnels. We discuss a number of primary and backup fixation strategies as well as all commonly used autografts (bone-patellar tendon-bone, hamstring, and quadriceps tendon). Additionally, we provide a brief overview on address intra-operative graft contamination citing currently available evidence.

## Introduction

Anterior cruciate ligament (ACL) injury is extremely common, with over 200,000 injuries per year in the United States. Of these, approximately 75% are treated surgically within the first year after injury [1], A significant proportion of these cases are performed by low-volume surgeons [2, 3]. ACL reconstruction is a technically demanding procedure, and classically the main reason for failure of surgical management is from technical error [4]. Low-volume surgeons may be more likely to encounter intraoperative difficulties [5]. Additionally, given significant variability in arthroscopic case volume during surgical training, it is possible that some lower-volume surgeons may not have been exposed to intraoperative error-recovery techniques during training [6]. Understanding and implementing effective intraoperative error recovery techniques are vital for ensuring patient safety, minimizing complications, optimizing outcomes.

A number of articles have been recently published discussing management techniques for intraoperative difficulties, including techniques for graft management and tunnel blowout [7–10]. However, there have been no unified reviews detailing intraoperative error recovery options for the low-volume surgeon or orthopedic surgery trainee. The goal of this review is to provide a practical, evidence-based guide for intraoperative recovery of common challenging scenarios that can be encountered during ACL reconstruction surgery.

## General Principles

### Femoral Tunnel

Femoral tunnel creation is perhaps the most important step of the ACL reconstruction procedure. Femoral tunnel positioning, angle, and length can lead to intra-operative difficulties and significantly impact the success rates of the procedure [11–13]. Because the target zone for anatomic, single-bundle ACL reconstruction leaves only a 1–2 mm posterior wall, an excessively posteriorly positioned femoral tunnel can violate the posterior cortex – commonly known as a posterior wall blowout [14]. This can complicate graft fixation necessitating a change in the operative plan [15]. In contrast, an excessively anteriorly placed femoral tunnel can lead to persistent rotational instability or graft impingement within the notch [9]. Another possible error during femoral tunnel drilling is lateral wall penetration if suspensory fixation is planned [16]. In the event of femoral tunnel issues, surgeons should be aware of techniques that enable recovery from these complications to allow successful completion of the case while optimizing patient outcomes (Table 1). The following sections describe intraoperative recovery techniques and the data behind them.

**Table 1:** Femoral tunnel error recovery techniques

	Lateral Wall Intact	Lateral Wall Compromised
Posterior Wall Intact	Any desired fixation	• Interference fixation
	• Suspensory fixation (any method)	• Screw post suspensory fixation
	• Interference fixation (any method)	• Extended cortical button (depending on size of lateral wall perforation)
Posterior Wall Compromised	Suspensory fixation (any method)	Screw post suspensory fixation

#### Posterior Wall Blowout

Posterior wall blowout can compromise graft fixation in the femoral tunnel; specifically, interference fixation relies on an intact tunnel to compress the graft and achieve appropriate healing. Disruption of the posterior wall can compromise this. Posterior wall violations that do not extend beyond 5 mm from the intra-articular tunnel aperture enable surgeons to proceed with planned interference screw fixation or suspensory fixation, though even minimal posterior wall blowouts can complicate graft fixation depending on the integrity of the remaining tunnel. Thus, surgeons should only continue with planned fixation if the minimal posterior blowout is recognized early and when the tunnel integrity can be fully evaluated [9]. To address early blowout, surgeons may redirect the reamer slightly anteriorly, ream the tunnel to a greater depth, and continue with planned fixation [15]. However, in scenarios where posterior wall blowouts extend further than 5 mm from the tunnel entrance or the rest of the tunnel wall is structurally compromised, other error recovery techniques should be considered.

#### Posterior Wall Blowout with an Intact Lateral Wall

**Fig. 1:**
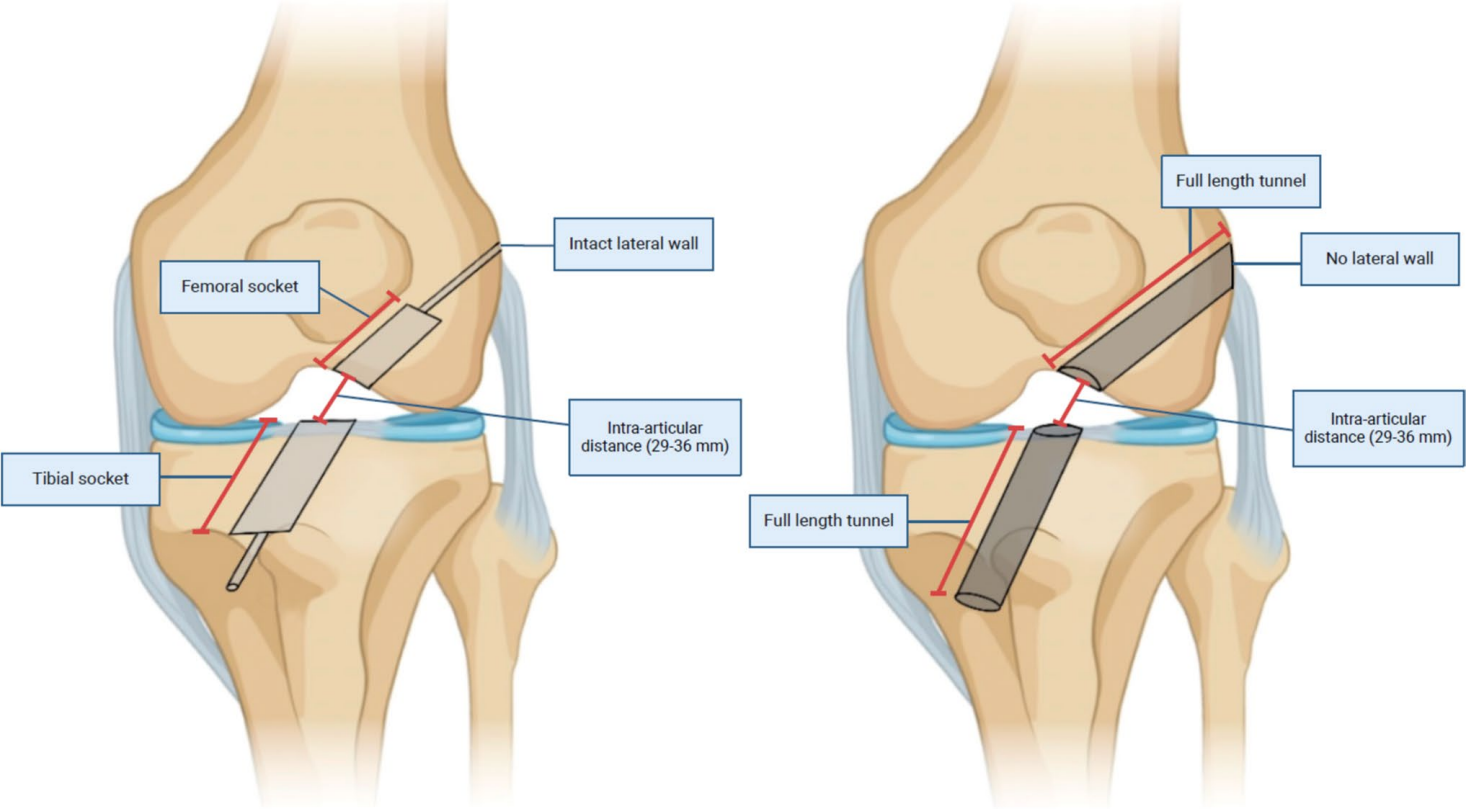
Figure 1a demonstrates femoral and tibial sockets with intact lateral wall on the femur and anterior wall on the tibia. Generally, sockets are utilized to allow for suspensory fixation, although femoral-sided interference screw fixation is still possible. When sockets are originally planned on the femur or tibia and are drilled out all the way, the is termed lateral or anterior cortex violation, respectively, and leads to the creation of full-length tunnels as showing in Fig. 1b. Figure 1b demonstrates femoral and tibial full-length tunnels. This can be the original planned technique depending on a surgeon’s preference. Traditionally, interference screw fixation is utilized using this technique, but suspensory fixation with a screw post is possible as well

**Fig. 2:**
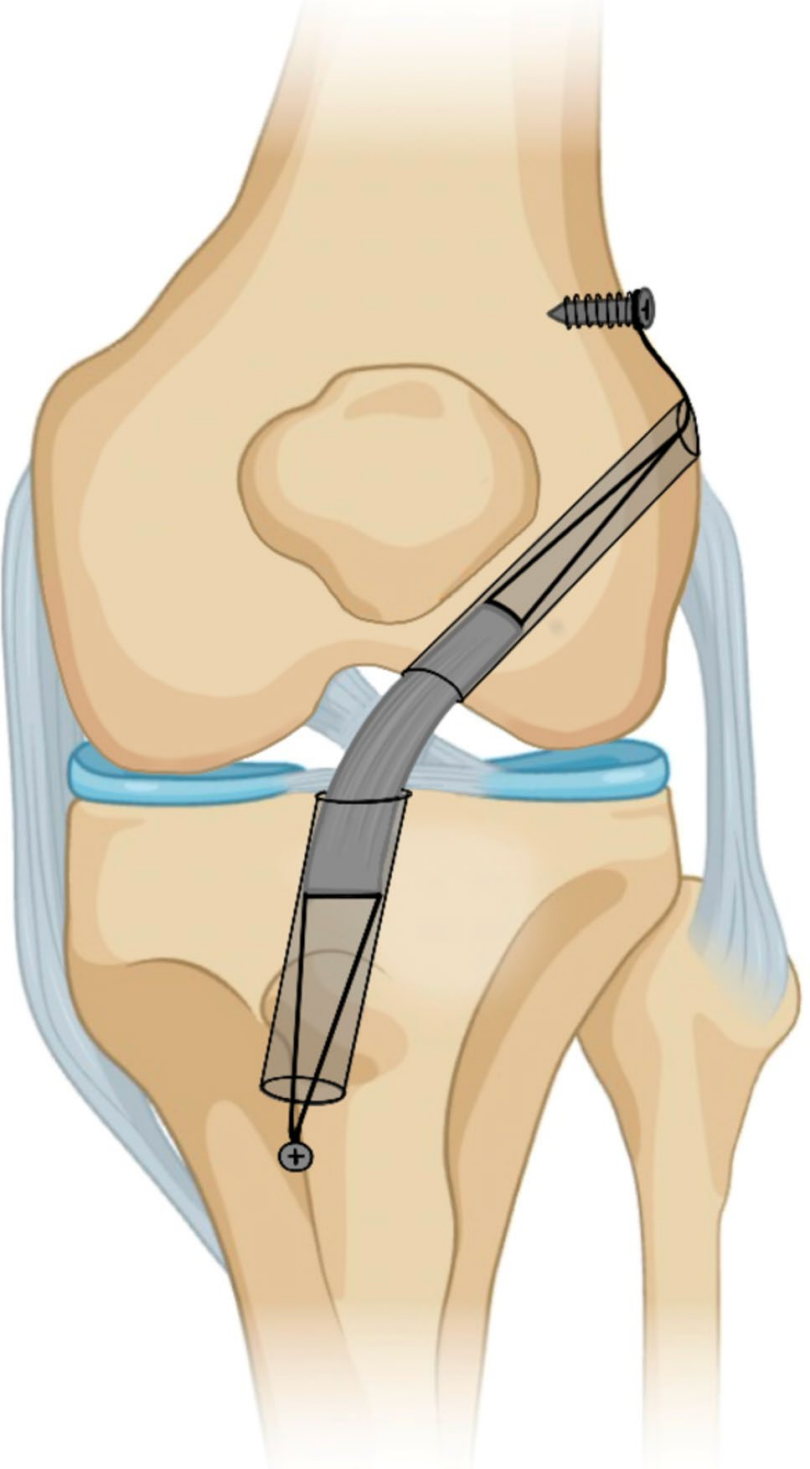
This demonstrates suspensory fixation with a screw post on both the femoral and tibial sides

When a substantial posterior wall blowout occurs but the lateral wall remains intact, numerous recovery techniques may be performed (Fig. 1a). If interference fixation is initially planned in these cases, fixation should be switched to suspensory fixation with either a cortical button or with screw and washer post [8, 17]. Suspensory fixation with a button can be used for both bone-tendon-bone (BTB) and soft tissue grafts but requires an intact lateral cortex. Suspensory cortical fixation with a screw and washer post can be used in cases with or without an intact lateral wall. This process involves creating a lateral incision over the distal lateral femur, dissecting down to the femoral tunnel, and securing the ACL graft by fixing a screw and washer post to the lateral femoral cortex (Fig. 2). These techniques are compatible with all grafts and only require equipment that is generally available during an ACL reconstruction procedure [8].

Another option for femoral ACL fixation with an intact lateral wall is a divergent tunnel technique—also known as a two-incision technique. A small incision is made along the lateral femur and a divergent tunnel is drilled with a more appropriate intra-articular aperture location [15]. Care should be taken when performing this technique to avoid overlap with the previously drilled intra-articular aperture; this may be difficult depending on the positioning of the previous tunnel. In cases with posterior wall blowout where the aperture must be moved anteriorly, there is biomechanical data showing that the suspended graft contacts the femoral tunnel aperture on the anterior distal aspect in lower degrees of knee flexion [18]. This may suggest that if the original tunnel is too posterior or proximal, even with some degree of overlap between the two intra-articular apertures, the graft will within the most anterior and distal tunnel. However, there are no clinical data to support these findings, and overlapping tunnels should be treated with caution.

Over-the-top fixation is another option if the femoral tunnel is unable to be salvaged; however this is a technically difficult option requiring additional posterior knee dissection which should be avoided by the low volume surgeon or those unfamiliar with the technique [9].

#### Posterior Wall Blowout without Intact Lateral Wall: Femoral Cortical Violation

Suspensory fixation with an extended cortical button and suspensory fixation with a screw and washer post can still be utilized even in scenarios where a posterior wall blowout is accompanied with the penetration of the lateral wall due to unintentional drilling of a full-length femoral tunnel (Fig. 1b) [8]. Typically, penetration of the lateral cortex impairs suspensory fixation with a standard cortical button because the button is too small to span the diameter of the lateral tunnel aperture for appropriate fixation following the violation [16]. In these scenarios, surgeons may use specialized extended length cortical buttons. However, these may not always be readily accessible intraoperatively. Even extended cortical suspension devices should be avoided when the lateral cortex is penetrated using a drill size that has a diameter greater than half of the size of the final planned cortical button [16]. Therefore, suspensory fixation with a screw and washer post may serve as an overall better alternative due to its flexibility with common tunnel sizes.

#### Isolated Lateral Cortex Penetration

Penetration of the lateral cortex may also occur in isolation. This is only considered an intraoperative error if the original intention was to drill a femoral socket with cortical suspensory fixation and the surgeon unintentionally creates a full-length tunnel by perforating the lateral wall. In such cases, surgeons may opt to switch to interference screw fixation [19]. Surgeons may also use suspensory fixation with a screw and washer post or extended cortical button as described in previous sections.

#### Anteriorly Placed Femoral Tunnel

Anterior placement of the femoral tunnel is the most common femoral tunnel malposition [20]. Anteriorly placed tunnels may be categorized as extremely anterior—defined as a femoral tunnel position that is greater than one tunnel diameter anterior—or slightly anterior—defined as a femoral tunnel position that is less than one tunnel diameter anterior. Extremely anterior and slightly anterior tunnels require different considerations and revision strategies. Extremely anterior femoral tunnels can be simply revised by drilling another tunnel in the anatomic position. This technique does not require the old tunnel to be addressed as long as the intra-articular tunnel apertures do not overlap. Care should be taken to drill the new tunnel in a divergent direction from the malpositioned tunnel. On the other hand, slightly anterior femoral tunnels are more difficult to manage. Surgeons should avoid drilling a new anatomically placed tunnel next to the original anterior tunnel as it would lead overlapping of the femoral apertures and a figure-of-eight shaped defect. This would complicate graft or bone block filling of the tunnel. In situations where the nonanatomic tunnel is placed less than 3 mm too anterior, the tunnel may be enlarged using a reamer and filled in with a large bone block with an attached allograft. The use of an allograft is recommended to mitigate harvest complications at the donor site when using larger bone blocks [20].

### Avoiding Femoral Tunnel Malposition

In general, avoidance of the above issues is the best approach when possible. The authors’ preference is to carefully evaluate the lateral wall at the time of femoral tunnel placement to identify the bifurcate ridge and intercondylar ridge, thereby outlining the native femoral footprint for the anteromedial and posterolateral bundles. An anatomic position should be mid-bundle and centered near the intersection of these ridges. After placement of a guide pin, a provisional 5–10 mm socket may be reamed, after which integrity of the back wall and position can be confirmed before further reaming a deeper socket. These steps may allow the surgeon to reliably confirm appropriate position and therefore avoid difficulties with fixation.

### Tibial Tunnel

Errors during tibial tunnel drilling are less-discussed in the literature. Malpositioning of the tibial tunnel can lead to inadequate restoration of knee stability or graft impingement. Additionally, problems during tunnel placement can cause difficulties with graft fixation as well. Tibial tunnel blowout during drilling or widening during attempted interference screw fixation may necessitate alterative graft fixation techniques (Table 2). These are discussed in the following section.

**Table 2:** Fixation options with tibial tunnel compromise

	Graft Lies Within Tunnel	Graft Extends Past Tunnel
Soft Tissue Graft	Screw post suspensory fixation	• Direct fixation (bone staple)
		• Suspensory fixation (screw post)
Bone Block Graft	Screw post suspensory fixation	• Direct fixation within a bone trough (bone staple, transfixation screw)
		• Suspensory fixation within a bone trough

#### Impingement

Anterior malposition of the tibial tunnel during ACL reconstruction can lead to graft impingement resulting in deleterious effects including knee instability, anterior knee pain, knee effusion, loss of knee extension, and graft failure [21, 22]. Theoretically, graft impingement may be addressed by performing notchplasty – a technique designed to increase the intercondylar notch space and reduce impingement [23]. However, clinical evidence of its efficacy is lacking, and several studies have reported regrowth of the notch following notchplasty [23–26]. While anterior tibial tunnel placement can contribute to impingement, this can also be the result of a malpositioned femoral tunnel – either too anterior or too proximal.

#### Tibial Tunnel Compromise

Tibial blowout occurs when the tibial tunnel is placed excessively anteriorly or the angle of drilling is too flat. This can result in disruption of the anterior aspect of the tibial tunnel compromising the anterior wall of the tunnel (tibial blowout) [27]. Because the proximal tibia is composed of soft metaphyseal bone, it is also possible to compromise the tibial tunnel during interference screw insertion by either stripping the tunnel or inserting the screw or dilator off-axis from the tunnel. This can widen the tunnel and impair graft fixation. If the anterior wall of the tibial tunnel is compromised, tunnel position should be carefully scrutinized. It is likely that the tunnel is too anterior and that graft impingement will occur if it is left as is. If the tunnel is significantly anterior, a new tunnel can be re-drilled with a higher angle to allow divergence of the new tunnel from the old tunnel, similar to management of a malpositioned femoral tunnel.

To achieve graft fixation with a compromised tibial tunnel (if interference fixation with a larger screw is unable to be achieved), it is important to note the amount of residual graft tissue. If the graft tissue extends past the length of the tibial tunnel, fixation with a bone staple can be utilized. This technique was traditionally used for tibial-sided graft fixation and is comparable in biomechanical strength to interference screw fixation [28]. If the graft does not extend past the tibial tunnel, suspensory fixation by fixing the graft sutures around a screw post construct in a method similar to that previously discussed for femoral-sided fixation can be utilized (Fig. 2) [8].

If the tibial tunnel is slightly anterior or posterior to an anatomic position after placement, the graft may be somewhat re-directed with the position of an interference screw for a soft tissue graft or by rotating the bone block and interference screw position for a bone-tendon graft. A slightly posterior tunnel may be overcome with posterior placement of an interference screw to push the graft more anteriorly. This technique, however, will only achieve slight differences in the graft, highlighting the need to ensure appropriate position of the tunnel before final reaming.

### Avoiding Tibial Tunnel Malposition

To avoid tibial tunnel malposition, the authors’ preference is to place a guidepin within the native ACL stump (if still present) and in line with the posterior border of the anterior horn of the lateral meniscus. Tunnel length can be identified prior to guidepin placement with most guide systems, and the drilling angle can be increased if the anticipate guide length appears short. After guide pin placement, the knee can be brought into full extension, to ensure that the pin placement will not interfere with reaching full extension prior to tunnel drilling. If a full tibial tunnel is reamed, the arthroscope can be placed within the tunnel after reaming to ensure that the bone is intact circumferentially prior to graft passage and fixation.

### Graft-Specific Issues

#### Hamstring Autograft

Graft diameter affects patient-reported outcomes, measures of knee stability, and graft failure following ACL reconstruction [29, 30]. BTB and quadriceps tendon harvests rarely pose issues with graft size due to the consistent ability to harvest the desired diameter. However, hamstring autograft harvests have a much greater size variability resulting in a higher prevalence of insufficient graft diameter [7]. Decreased hamstring graft sizes are predictors for early graft revision and correlate with higher revision rates in young patients [31]. Additionally, investigators have found that for every 0.5 mm increase in graft diameter for grafts larger than 7 mm, there is a 0.82 times lower likelihood of revision [32]. Surgeons should strive to harvest a hamstring autograft diameter of at least 8 mm to minimize the risk of graft failure and revision, especially in patients under 20 years old [7, 31]. Classically, hamstring autografts have been described as double folded semitendinosus and gracilis tendons, resulting in a quadrupled hamstring graft; however, this technique can often result in grafts of inadequate diameter [33]. Especially in younger patients of smaller stature, surgeons should be wary of the potential need for graft augmentation. Preoperatively, MRI measurements of the hamstring tendons can be reliably used to predict final quadrupled hamstring tendon graft diameter [34]. This can be utilized preoperatively in smaller stature patients to predict possible intraoperative issues with graft diameter and guide the preoperative graft-selection conversation. The following techniques provide possible solutions for inadequate hamstring graft diameter (Table 3).

**Table 3:** Solutions for inadequate hamstring graft diameter

Technique	Pros	Cons
Multiple (5-, 6- or greater) strand graft	• Provides larger diameter graft using native tissue	• Reduced final graft length
	• No additional harvest site morbidity	• May require a change in fixation technique (suspensory fixation on both tibial and femoral side)
Suture augmentation	• No additional tissue required	• Data limited to biomechanical studies – no clinical data
Allograft augmentation (hybridization)	• Allows significant flexibility in graft creation	• Conflicting clinical evidence – use with caution
	• No additional harvest site morbidity	• Additional consent needed to utilize allograft tissue
Quadriceps tendon augmentation	• Allows full autograft tissue when there is inadequate hamstring tissue or quadriceps tissue independently	• Data limited to biomechanical studies – no clinical data
		• Additional harvest site – increased morbidity

### Multiple Strand Construct Creation

After harvesting an insufficiently sized hamstring autograft, surgeons may create a 5-strand autograft construct to increase graft width and secure the construct using interference screw or suspensory fixation [7]. Typically, most surgeons utilize a 4-strand hamstring autograft configuration formed from doubling gracilis and semitendinosus tendons [31]. 5-strand hamstring autograft constructs consist of a doubled gracilis and tripled semitendinosus tendon and may be utilized to increase graft diameter to 8 mm or greater without reducing overall outcomes [35]. Studies have demonstrated that 5-strand autograft constructs of 8 mm or larger have no statistically significant differences in re-rupture incidences, clinical outcomes, and patient-reported outcomes in comparison to 4-strand autograft constructs of 8 mm or larger [36].

Furthermore, 6-, 7-, or 8-strand constructs are also conceivable if the gracilis and semitendinosus tendons are long enough. Some surgeons have utilized quadrupled, isolated semitendinosis grafts in all-inside repair constructs. Early results from these trials have shown non-inferiority to BTB autograft techniques [37]. Utilizing all-inside or double-suspensory reconstruction techniques, one can theoretically use a quadrupled semitendinosis graft in addition to a tripled or quadrupled gracilis graft depending on harvested tendon length. One previous investigation has demonstrated no difference in failure rates at 2-year follow-up with 5- or 6- strand hamstring grafts versus 4-strand grafts using double-suspensory fixation [38]. No data on additional stranded hamstring grafts exists. Assuming surgeon comfortability with both femoral and tibial-sided suspensory fixation techniques, 6-strand hamstring grafts are a feasible option to achieve a good final construct diameter.

When creating a multiple-strand hamstring graft, the number of strands in limited by the length of the harvested hamstring and the target length of the graft. The total graft length is the sum of the femoral side length, intra-articular length, and tibial side length. The length of the femoral and tibial tunnel is determined by the surgeon. Traditionally, minimum socket lengths of 20–25 mm on the femoral and tibial sides have been recommended, but studies with shorter femoral tunnel lengths (some below 15 mm) have been published demonstrating no inferior clinical outcomes [39, 40]. The intra-articular length is patient-specific, but generally varies between 29 and 36 mm [39, 41]. Thus, the minimum target graft length should be between 60 and 80 mm depending on surgeon preference and fixation technique.

### Hybridization

Hybridization is another technique that can increase the diameter of insufficiently sized hamstring autografts by supplementing with allograft tissue. However, the effectiveness of this technique is unclear. While it would seem that adding tissue would lower the likelihood of graft failure and improve the structural properties of a smaller autograft, allografts may mature at a slower rate, leading to the uncertainty of the effectiveness of hybrid grafts. The current research has demonstrated mixed outcomes for hybridization [7]. In one study, hybrid grafts demonstrated higher odds of rupture than autografts in adolescents [42]. Furthermore, even hybrid grafts with large diameters have displayed significantly higher graft failure rates than insufficiently sized hamstring autografts in adolescent patients [43]. On the other hand, several systematic reviews have found variable graft failure rates without a clear pattern that favors the use of autografts over hybrid grafts as well as no significant differences between graft failure rates, patient-reported outcome scores, objective knee measures of stability, and revision rates between autografts and hybrid grafts [44, 45]. Given the conflicting evidence, surgeons should be cautious when utilizing hybridization as a recovery technique to address inadequate hamstring tendon graft diameter, especially in young, active patients [42, 43]. Our preference is to utilize 5- or 6-strand grafts, when at all possible, over a hybrid autograft-allograft construct for these reasons.

### Augmentation with Suture Brace

Recently, surgeons have considered augmenting small-diameter autografts with a nonresorbable suture. The theory is that a suture would reinforce small-diameter autografts and strengthen the overall construct [7]. The limited in vitro research has demonstrated that high-strength suture tape augmentation with small-diameter hamstring autografts significantly increased failure loads [46]. Currently, there are no significant in vivo or human studies assessing the outcomes of augmenting small-diameter hamstring autografts with suture. While the in vitro findings are promising, our preference would be to utilize multi-strand graft or a hybrid construct over a smaller graft with suture augmentation alone.

### Augmentation with Quadriceps Tendon

Small-sized hamstrings autografts may also be augmented with ipsilateral quadriceps tendon autografts. Quadriceps augmented hamstring grafts have demonstrated similar biomechanical properties to sufficiently sized hamstring autografts in vitro; no statistically significant differences have been found in tensile failure load, energy absorbed, stiffness, and displacement between quadriceps augmented hamstring graft constructs and sufficiently sized hamstring autografts [47]. This suggests that quadriceps tendon autograft augmentation may be a viable method for increasing hamstring graft diameter, especially when the patient nor the surgeon want to utilize allograft to supplement an undersized hamstring autograft. It is important to consider, however, that there is limited in vivo data on the outcomes of this technique.

### Premature Truncation of Hamstring Graft

Premature graft truncation is another complication that may arise during hamstring harvest. This occurs during harvest from an anterior approach usually from improper separation of the hamstring tendon from the fascial bands and accessory insertions before tendon stripping [48]. This results in shorter grafts and may require alternative harvesting techniques. A minimally invasive posterior hamstring harvest is an alternative technique that mitigates complications associated with the anterior approach and premature truncation of hamstring tendons, and it can be utilized to salvage the remainder of the hamstring tendon if it is truncated prematurely during an anterior harvest [49, 50]. This technique can be used to harvest medial hamstring, semitendinosus, or gracilis tendons.

Minimally invasive posterior hamstring harvests begin with a 2 to 3 cm longitudinal incision proximal to the popliteal crease, followed by dissection down to the hamstring tendons. The targeted tendon is located and clamped, and the surrounding soft tissue, fascia, and accessory attachments are dissected until there are no adhesions. The tendon stripper is then applied to the tendon. It is important to note that the location of the semitendinosus tendon may vary. The tendon is typically positioned most laterally; however, occasionally, the semimembranosus tendon is oriented most lateral. To distinguish between the two, the semitendinosus tendon is palpable distally at the pes anserine (which may not be identifiable if the tendon has already been released), while the semimembranosus is attached to the proximal, posterior, and medial tibia distally [50].

#### Quadriceps Tendon Autograft

The use of quadriceps tendon (QT) autograft has gained popularity in recent years as an alternative to BTB and hamstring autografts [51]. QT grafts have demonstrated similar outcomes to these traditional graft options while showing lower levels of donor site morbidity as comparted to BTB autograft [52]. However, because of the relatively new popularity of this graft options and the learning curve associated with it, surgeons adopting this technique may be more likely to encounter intraoperative graft issues that they are not prepared to address [53, 54].

The QT graft is harvested as a single bundle; as such, graft length and diameter are strongly dictated by the patient’s anatomy. When patients are smaller in stature, it is important to be aware of the possibility that tendon graft may be less than 8 mm in diameter or 65 mm in length, the generally accepted minimum graft dimensions [53, 55, 56]. Although these graft sizes are generally attainable in even pediatric patients, it is important to evaluate patient size and preoperative MRI prior to surgery to highlight patients who should be consented for graft augmentation techniques [55]. Specifically, when patients approach 60 inches or less in height (approximately 152 centimeters), tendon length will often be around the 65 mm mark [57]. For graft diameter, 6.7 mm thickness on MRI is 97.4% sensitive for a minimum 8 mm graft diameter [55].

### Inadequate Graft Length

Intraoperatively, when inadequate graft length from the proximal patellar edge to the quadriceps tendon musculotendinous junction is encountered, there are a number of techniques to address this. First, ensure that measurement is occurring slightly lateral to the midline of the patella – this will allow for measurement of the maximum length of the QT prior to the musculotendinous junction, which occurs approximately 60% of the distance away from the medial edge of the patella [58]. If minimal additional length is required (< 1 cm) a periosteal sleeve up to 2 cm in length and the same width as the QT can be elevated off the anterior surface of the patella. This can then be folded over length-wise and incorporated into soft-tissue fixation, adding additional length to the final graft. If more additional length is required (> 1 cm) a bone block can be harvested [59, 60]. Harvest of the bone block is similar as with the BTB procedure; however, care should be taken to harvest less than 50% of the patellar depth from the central region of the patella, as there is the potential for a higher rate of postoperative patellar fracture than with BTB procedures [53, 61]. The addition of these intraoperative recovery techniques to a surgeon’s repertoire should allow for adequate graft length to be obtained in all but the rarest of cases.

### Inadequate Graft Diameter

Graft diameter of less than 8 mm has been associated with a greater risk of retear in hamstring autografts [29, 62]. Although these findings have not yet been borne out in quadriceps autografts, similar cutoffs in graft diameter have been proposed [55]. Regardless of a surgeon’s personal cutoff for graft diameter, it is important to have a recovery option in case the harvested tendon diameter is insufficient. For BTB grafts this is not a significant issue as the size of the graft is not as variable between patients (generally the central one-third of the tendon and approximately 10 mm in width as a standard), and the limited investigations on the relationship between graft diameter and retear have not found a significant relationship [62]. To minimize the risk of inadequate graft diameter, a QT graft of 9–10 mm in width should be harvested; based on anatomic studies, this graft width, when centered slightly lateral to the midline of the patella should provide an appropriate graft diameter and length [58]. However, technical errors during harvest of aberrant patient anatomy may still sometimes lead to a situation when inadequate quadriceps tendon graft diameter is encountered. In this case, augmentation with a single hamstring tendon autograft or allograft may be considered [47]. Additionally, a synthetic graft augmentation with high-strength suture can be considered. This technique can slightly improve the graft diameter, and has been shown to potentially reduce graft elongation and damage in animal models; however, long-term clinical data are lacking [63, 64].

#### Bone-Patellar Tendon-Bone Autograft

BTB autografts have historically been the gold standard for ACL reconstruction and offer the benefits of bone-to-bone healing. Given the long length and width of the patellar tendon, there is generally sufficient graft length and width. However, these grafts present the unique problem of graft-tunnel mismatch, a situation in which the length of the graft does not appropriately match the length of the combined intra-articular length and femoral and tibial tunnel lengths. The problem of graft-tunnel mismatch occurs almost exclusively when the BTB graft is too long, and should be suspected if patella alta is noted preoperatively. There are a number of techniques available to address this problem including graft twisting, supplemental screw post or bone staple fixation, doubling the bone block back over tendon graft, and recessing the femoral side in a deeper tunnel. In a recent survey of mostly high-volume, sports fellowship-trained orthopedic surgeons, no consensus could be found in the techniques used in management of graft-tunnel mismatch [10]. These surgeons estimated that the severity of graft-tunnel mismatch was generally less than 10 mm. We will provide some options for preventing and addressing graft-tunnel mismatch and discuss the two most highly-reported techniques by experienced surgeons: femoral tunnel recession and screw post fixation +/- bone trough (Table 4).

**Table 4:** Solutions for Graft-Tunnel Mismatch

Technique	Amount of Correction Obtainable	Steps and Pearls
Femoral Tunnel Recession	Determined by length of femoral tunnel minus length of bone block	• Full length femoral tunnel with interference fixation is required to maximize corrective power
		• Second incision over lateral femur is required to allow outside-in interference screw placement
		• Mismatch should be identified prior to femoral-sided fixation
Fixation within Trough	Unlimited	• Fix one end of graft (general femur) as planned and pull other end of graft out to length to determine the location of the bone trough
		• Bone block must be fully free of the tunnel for this technique
Graft Rotation	Up to 12 mm	• Rotation of up to 540 degrees has been investigated[73]
		• Most surgeons rotate the graft less
		• Amount of correction correlates with degrees of rotation utilized
Bone Block Flipping	Based on length of bone block (theoretically both sides of the graft can be flipped to allow twice the correction)	• Bone block flipped 180 degrees
		• Standard interference fixation
Free Bone Block	Unlimited	• Remove bone block, pull soft tissue to length, insert bone block into tunnel to compress soft tissue graft, then proceed with standard interference fixation
Bone Block Removal	Unlimited	• Remove bone block and proceed with soft tissue fixation (interference or suspensory)

### Setting Tibial Tunnel Angle

An important step to avoid graft-tunnel mismatch is to choose an appropriate tunnel length. Specifically, on the tibial side the “N + 10” rule can provide guidance on the appropriate angle of the tibial tunnel to drill. This rule dictates that a steeper tibial angle (and thus longer tibial tunnel) should be drilled for longer grafts. It was validated in a cadaveric study showing that by measuring the tendinous portion of the graft and adding 10 degrees, an appropriate length tibial tunnel was able to be created in 90% of cases [65].

### Femoral Tunnel Recession

The primary strategies to address graft-tunnel mismatch should either address the length of the tunnels (femoral and/or tibial) or the length of the graft, as the intra-articular length is not able to be adjusted freely. The survey study by Saltzman et al. identified femoral tunnel recession as the primary technique utilized when graft-tunnel mismatch is < 10 mm, the most commonly reported amount [10].

**Fig. 3:**
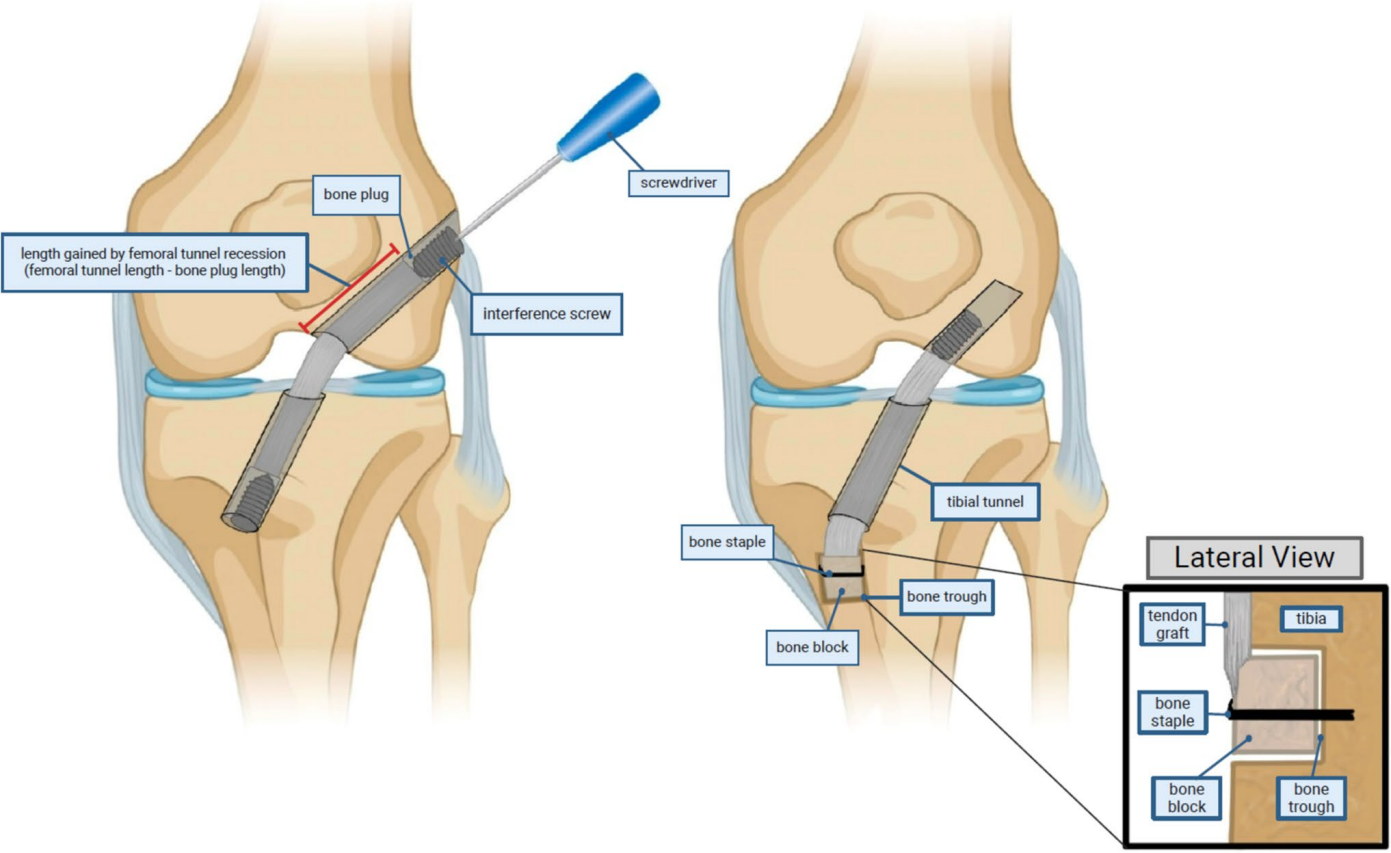
Figure 3a demonstrates femoral tunnel recession on the femoral side. In this case, the interference screw should be placed from outside-in. This allows a gain in tunnel length that is equal to the full length of the femoral tunnel minus the length of the bone block compared to fixing with an interference screw from the intra-articular aperture (inside out). Figure 3b demonstrates a bone trough with staple fixation. A trough is created in the tibial bone to accommodate the bone plug on the graft; this allows essentially unlimited correction when graft-tunnel mismatch occurs

With femoral tunnel recession, the femoral socket can be drilled deeper, or the lateral cortex can be penetrated to create a full-length femoral tunnel depending on the amount of femoral recession desired. While creation of a full-length tunnel can often provide approximately 30 mm of tunnel length – which is often enough to obviate any issues with graft-tunnel mismatch – it may necessitate a change in graft fixation technique (Fig. 3a). If cortical button suspensory fixation of the femoral tunnel is desired, care must be taken to avoid penetrating the lateral cortex when recessing the femoral tunnel; however, if this occurs suspensory fixation can still be achieved using screw post fixation [8]. Although no large head-to-head clinical trials comparing suspensory to interference fixation have been conducted, small-scale imaging and clinical investigations have shown adequate bone-to-bone healing with suspensory fixation [66, 67]. Theoretical concerns about graft abrasion on the femoral aperture with a recessed femoral bone graft have not been borne out in clinical investigations [68].

If interference fixation is an option, outside-in fixation can be performed after creation of a full-length femoral tunnel to maximize the amount of graft incorporated into the femoral tunnel; this requires cutting down to the lateral cortical aperture and inserting an interference screw directed towards the joint (similar to the standard technique of inference screw fixation on the tibial side). After fixation, any proud bone block can be trimmed. Care should be taken to maintain at least 20 mm of bone block in the tunnel, as shorter bone blocks have demonstrated failure in a biomechanical investigation [69]. Utilizing outside-in interference fixation of both the femoral and tibial tunnels should essentially obviate graft tunnel mismatch in all but the most difficult of cases.

### Bone Block Fixation in Trough

The most common fixation technique identified for larger amounts of graft-tunnel mismatch in the survey study by Saltzman et al. was screw post +/- bone trough creation [10]. This can be done on either the femoral or tibial side. The graft is fixed in the opposite tunnel (usually the femoral tunnel) and pulled to appropriate tension. The location for the desired bone trough is then identified (usually on the tibial side) by pulling the graft out to length and laying it flat on the bone. The trough is created to accommodate the bone block, and the bone block is seated within the trough (Fig. 3b). Fixation at this point can be done with a number of techniques, including screw post fixation, staple fixation, or with a screw through the bone block [8, 28]. This technique maintains the benefits of bone-to-bone healing and is flexible enough to account for even the most severe cases of graft-tunnel mismatch.

### Alternate Methods

Other less commonly-utilized alternatives for graft-tunnel mismatch include free bone block, graft twisting, bone block removal (with isolated soft tissue fixation), and flipping the bone plug [10]. These techniques, though less utilized, can be alternative solutions to graft-tunnel mismatch, and surgeons performing ACL reconstruction should familiarize themselves with these techniques which have been generally shown to provide acceptable graft fixation [7]. Regardless of method used, at least 12.5 mm of bone block should be retained within the tunnel if interference fixation is utilized [70].

### Intraoperative Graft Contamination

In the case of intraoperative graft contamination, the options are to decontaminate and reuse the original graft, utilize an alternative harvest site, utilize an allograft, or abort the procedure. Alternative graft harvest and allograft utilization require preoperative patient consent. Although this is an extremely difficult intra-operative decision, one previous survey study provides important guidance on this topic [71]. In this study, a questionnaire was sent to fellowship-trained sports medicine surgeons inquiring about episodes of ACL graft contamination, treatment preferences, and surgeon-reported clinical outcomes.

In this study, 71% of sports fellowship-trained surgeons who responded experienced graft contamination at some point in their careers, and in 75% of cases the contaminated grafts were cleaned and the procedure went forward as planned [71]. Graft cleansing protocols varied widely, but most utilized some variation of cleansing with a chlorhexidine gluconate, antibiotic, or povidone-iodine solution with no surgeon-reported cases of postoperative infection.

No clinical studies have been conducted evaluation rate of postoperative infection after graft decontamination. However, a number of basic science studies have been performed [7, 72]. A recent meta-analysis of these investigations has demonstrated a contamination rate (as judged by positive cultures) of approximately 45% after dropping the graft on the operating room floor [72]. While a 4% chlorhexidine solution wash appeared to be most efficacious method of decontamination, it still led to a culture positivity rate of 2.3% after treatment (versus approximately 10% with other treatment methods).

One proposed cleansing protocol is a multistep protocol involving removal of all material from the graft (suture materials) followed by a 15-minute soak in 2% chlorhexidine solution with additional soaks in antibiotic and/or povidone-iodine solutions followed by a saline rinse prior to implantation. Ultimately, no ideal treatment algorithm exists, but the data suggests that chlorhexidine (either at a 2% or 4% concentration) is the most efficacious decontamination agent and should be incorporated into any cleaning protocol that is utilized.

## Conclusion

ACL reconstruction is a technically challenging operation, especially for trainees and low-volume surgeons. This procedure has multiple steps during which intraoperative errors can occur, and it is imperative that surgeons know error management techniques to recover from any of these potential problems. Difficulties can be encountered during tunnel preparation – an essential part of the procedure – or can be graft-specific in nature. While modern graft fixation techniques provide increased flexibility and ease of use for surgeons, it is imperative for surgeons to understand alternative fixation techniques to adapt to intraoperative challenges.

## Data Availability

No datasets were generated or analysed during the current study.
